# A novel liver metastasis-correlated protein of pancreatic neuroendocrine neoplasm (PanNEN) discovered by proteomic analysis

**DOI:** 10.18632/oncotarget.25110

**Published:** 2018-05-11

**Authors:** Mitsuhiro Shimura, Masamichi Mizuma, Tatsuyuki Takadate, Yasutake Katoh, Takashi Suzuki, Masahiro Iseki, Tatsuo Hata, Shuichi Aoki, Yukie Suzuki, Naoaki Sakata, Hideo Ohtsuka, Hiroki Hayashi, Takanori Morikawa, Kei Nakagawa, Fuyuhiko Motoi, Takeshi Naitoh, Kazuhiko Igarashi, Hironobu Sasano, Michiaki Unno

**Affiliations:** ^1^ Department of Surgery, Tohoku University Graduate School of Medicine, Aobaku, Sendai 980-8574, Japan; ^2^ Department of Biochemistry, Tohoku University Graduate School of Medicine, Aobaku, Sendai 980-8574, Japan; ^3^ Center for Regulatory Epigenome and Diseases, Tohoku University Graduate School of Medicine, Aobaku, Sendai 980-8574, Japan; ^4^ Pathology and Histotechnology, Tohoku University Graduate School of Medicine, Aobaku, Sendai 980-8574, Japan; ^5^ Department of Pathology, Tohoku University Graduate School of Medicine, Aobaku, Sendai 980-8574, Japan

**Keywords:** PanNEN, liver metastasis, CNPY2, ANXA6, proteomics

## Abstract

The aim of this study was to identify novel liver metastasis-correlated proteins of PanNEN by proteomics to compare pancreatic tumor (PT) with paired metastatic liver tumor (LT). Of 118 surgical cases with PanNEN, 7 cases with formalin-fixed paraffin-embedded (FFPE) tissues of both PT and paired LT were evaluated by proteomics. Tumor cells were selectively collected from FFPE tissues by laser capture microdissection. A total of 3,722 proteins were detected from extracted peptides by mass spectrometry-based shotgun analysis. Selection of the candidate proteins expressed differently between PT and LT were performed by semi-quantitative comparison *in silico* and confirmation with immunohistochemistry. We focused on ANXA6, CNPY2, RAB11B and TUBB3, all of which had higher expressions in LT. In all surgical cases with FFPE samples, liver recurrence-free survival (RFS) was evaluated in correlation to the expression of the candidate proteins in PT by immunohistochemistry. Liver RFS was significantly poorer in CNPY2 positive patients than in negative patients (10-year liver RFS; 39.8% vs. 92.3%, p = 0.012). Also, liver RFS tended to be poorer in ANXA6 positive patients than in those who were negative (10-year liver RFS; 51.4% vs. 95.0%, p = 0.099). In the multivariate analysis, the independent predictors of liver RFS were CNPY2 positivity (HR: 6.19, 95 % CI: 1.47–42.79, p = 0.011) and tumor size ≥ 42 mm (HR: 4.63, 95 % CI: 1.03–23.23, p = 0.045). In conclusion, CNPY2 is a novel liver metastasis-correlated protein of PanNEN.

## INTRODUCTION

Neuroendocrine tumors (NET) are relatively rare neoplasms. However, the number of patients with NET has recently been increasing all over the world [1, 2]. In Japan, the incidence of pancreatic neuroendocrine neoplasm (PanNEN) has been reported to be increasing, showing a 1.2-fold increase from 2005 to 2010 [3]. Among NETs of various organs, the prognosis of PanNEN is poorer [3]. Liver metastasis, found in 42.0% at the initial diagnosis, is one of the poor prognostic factors of PanNEN [3]. The molecular mechanism, promotors, and inhibitors of liver metastasis of PanNEN are mostly unknown. Liver metastasis of PanNEN is generally treated with liver resection, chemotherapy, peptide receptor radionuclide therapy (PRRT), and so on. Several reports have shown that the five-year survival rate after liver resection for PanNEN is 61–76%, and the liver recurrence rate is 54–94% at 5 years after prior liver resection [4–7]. Thus, it is difficult to obtain cure by hepatectomy for liver metastasis of PanNEN. Also, the efficacy of non-surgical treatment for metastatic PanNEN is limited. Therefore, since new treatment strategies are required to improve the prognosis of PanNEN with liver metastasis, it is very important to identify novel liver metastasis-correlated molecules of PanNEN.

Recently, novel biomarkers or target proteins of metastasis in several malignancies, such as pancreatic cancer, bile duct cancer, colorectal cancer, and lung cancer, have been identified by proteomics using clinical samples [8–14]. Prognostic or metastasis-correlated proteins have been examined by proteomic analyses between the primary tumor and metastatic tumor in colon or lung cancer [13, 14]. However, only two studies on the proteomics of PanNEN have been reported. One was a proteomic analysis between insulinoma with and without lymph node metastases [15], and the other one between the tumor tissue of insulinoma and normal tissue [16]. There has been no study of proteomic analysis comparing the primary tumor of PanNEN with liver metastasis. The aim of the present study was to elucidate novel liver metastasis-correlated proteins of PanNEN by proteomic analysis to compare the primary tumor with the paired liver metastasis.

## RESULTS

### Discovery stage

#### Protein identification by shotgun proteomics and semi-quantitative comparison

**Figure 1:**
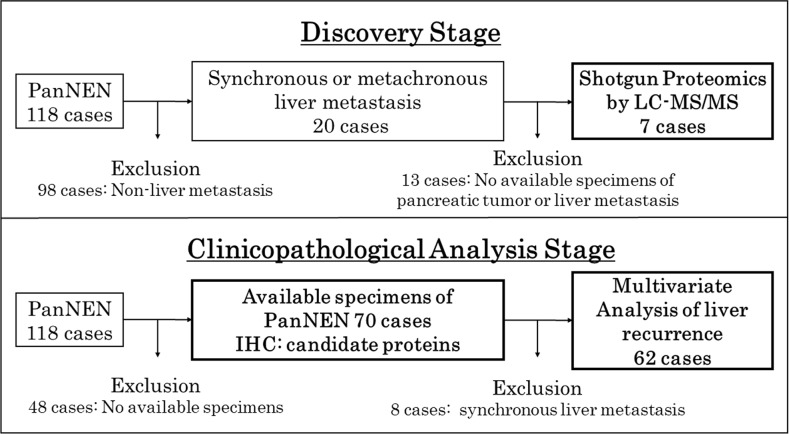
Study flow chart of the sample selection The study was performed in two stages, a discovery stage for shotgun proteomics by LC-MS/MS and a clinicopathological analysis stage for analysis by IHC and multivariate analysis of liver recurrence.

**Figure 2:**
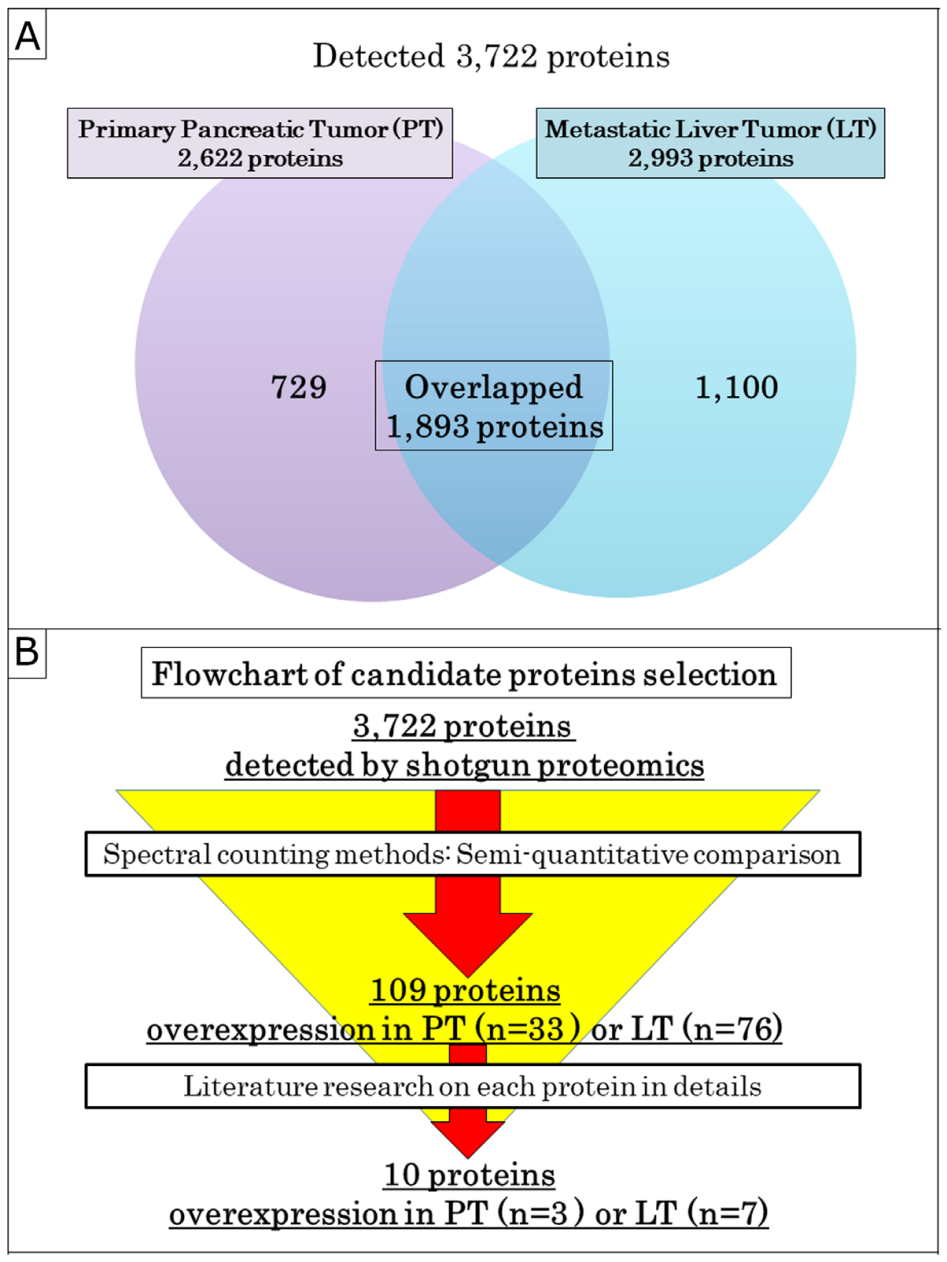
Selection of the candidate proteins **(A)** Venn diagram of proteins detected by shotgun proteomics. **(B)** Flowchart of the candidate proteins selection. All of the 3,722 proteins identified by shotgun proteomics were semi-quantitatively analyzed by the spectral counting method and were narrowed down to 109 proteins. Next, 10 candidate proteins, including 3 and 7 proteins overexpressed in PT and LT, respectively, were selected from among 109 proteins by a literature review on biological role and relevance of malignant disease.

Both primary pancreatic tumor (PT) and paired metastatic liver tumor (LT) samples of 7 cases with PanNEN were investigated by proteomic analysis to determine differences between their protein expressions (Figure 1 and Table 1). A total of 3,722 proteins, including 2,622 proteins in PT and 2,993 in LT, were identified by shotgun proteomics (Figure 2A). Seven hundred and twenty-nine proteins (19.6%) were identified in PT alone, while there were 1,100 proteins (29.6%) in LT alone. 1,893 proteins (50.8%) overlapped. All of the 3,722 proteins identified by shotgun proteomics were compared semi-quantitatively using spectral counting methods (Figure 2B). Based on Rsc >1 or <-1, and statistical significance (p < 0.05 by *G*-test), 33 and 76 proteins were overexpressed in PT and LT, respectively, were selected. Of these proteins, we focused on 3 and 7 proteins overexpressed in PT and LT, respectively, as candidate proteins potentially associated with malignant disease, referring to previous reports, and so on (Table 2).

**Table 1:** Patient characteristics examined by proteomics

Case	Age	Gender	LT	WHO 2017 Grade	Ki-67 (%)	Function type	Primary tumor diameters (mm)	RFS (months)	Prognosis (OS, months)
1	53	Male	synchronous	G2	5.0	NF	14	-	Alive (89.3)
2	49	Female	synchronous	G3	30.5	NF	70	-	Alive (73.1)
3	63	Female	synchronous	G3	25.0	NF	46	-	Alive (55.2)
4	69	Male	synchronous	G2	8.0	NF	75	-	Dead (72.7)
5	53	Male	metachronous	G1	2.7	Gastrinoma	50	25.4	Dead (87.0)
6	75	Male	metachronous	G2	11.1	NF	150	24.6	Dead (80.0)
7	25	Male	synchronous	G3	24.5	NF	40	-	Dead (77.7)

Abbreviations: NF: non-functioning; RFS: recurrence-free survival; OS: overall survival.

**Table 2:** List of the candidate proteins selected by shotgun proteomics and semi-quantitative comparison

UniProt Accession Number	Official Symbol	Fold Change	Function by Gene Ontology Annotation Database	Related pathway by KEGG PATHWAY Database
Selected overexpressed proteins in pancreatic tumor				
P62491	RAB11A	2.8	GTP binding; GTPase activity; Microtubule binging; Myosin V binding; Protein binding	Endocrine and other factor-regulated calcium reabsorption; Endocytosis; Pancreatic secretion: Vasopressin-regulated water reabsorption
F5H7S3	TPM1	6.6	Actin biding; Cytoskeletal protein binding; Structural constituent of cytoskeleton; Structural constituent of muscle	MicroRNAs in cancer; Cardiac muscle contraction; Adrenergic signaling in cardiomyocytes etc.
Q15836	VAMP3	3.9	Protein binding; SNAP receptor activity; SNARE binding	Phagosome; SNARE interactions in vesicular transport
Selected overexpressed proteins in liver tumor				
P08133	ANXA6	2.2	GTP binding; calcium-dependent phospholipid binding; calcium-dependent protein binding etc.	No hits
Q9Y2B0	CNPY2	2.2	Protein binding	No hits
P68431	HIST1H3A	2.1	Cadherin binding; histone binding; nucleosomal DNA binding; protein binding	Transcriptional misregulation in cancer; Alcoholism; Systemic lupus erythematosus
Q15691	MAPRE1	2.3	RNA binding; cadherin binding; identical protein binding; microtubule plus-end binding; protein C-terminus binding etc.	No hits
Q15907	RAB11B	2.1	GDP binding; GTP binding; GTPase activity; cadherin binding; myosin V binding; protein binding	Endocytosis; AMPK signaling pathway; Vasopressin-regulated water reabsorption
P67812	SEC11A	3.2	Peptidase activity; serine-type peptidase activity	Protein export
Q13509	TUBB3	5.1	GTP binding; GTPase activity; Protein binding; Structural constituent of cytoskeleton	Phagosome; Gap junction; Pathogenic Escherichia coli infection

#### Validation of protein expression by immunohistochemistry

**Figure 3:**
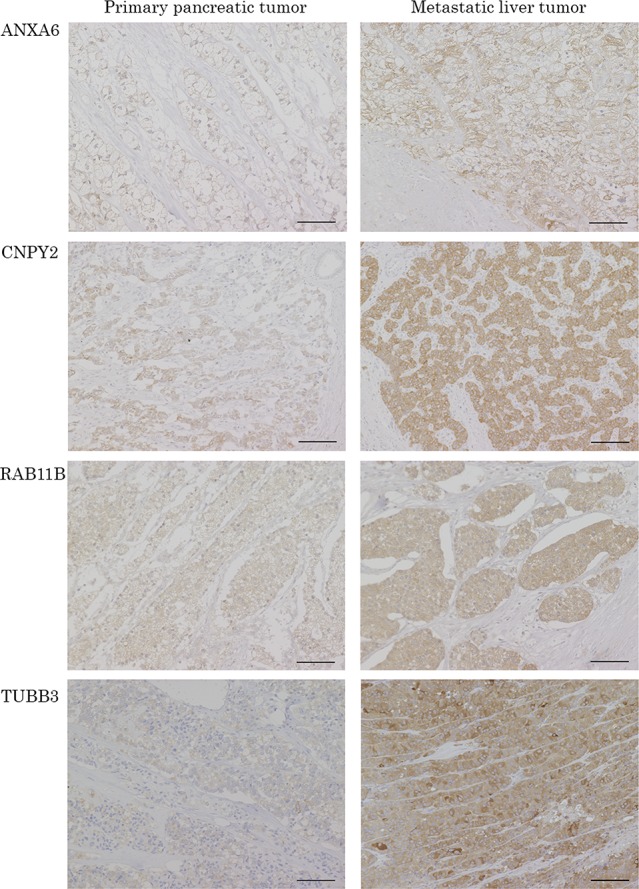
Representative pictures of IHC for the candidate proteins in PT and paired LT The protein expressions of ANXA6, CNPY2, RAB11B and TUBB3 were confirmed in the patients evaluated with proteomics by IHC. ANXA6, CNPY2, RAB11B and TUBB3 are IHC images of patient NO.6, 4, 2 and 3, respectively. Scale bars indicate 100 μm.

In order to confirm the results of proteomic analysis, the expressions of 10 candidate proteins in samples from the discovery stage were evaluated by immunohistochemistry (IHC). Only Histone H3.1 (HIST1H3A) was expressed at the nuclear level, and the others were at the cytoplasm and/or cell membrane level. One protein was not immunoreactive, but the others, which were positive in the tumor cells, could be evaluated. Comparing the protein expressions between the tumors and background normal tissue, or PT and paired LT, Annexin A6 (ANXA6), the canopy FGF signaling regulator 2 (CNPY2), Ras-related protein Rab-11B (RAB11B) and Tubulin beta-3 chain (TUBB3) were the most compatible with the results of the proteomic analysis, and were expressed higher in LT than in PT (Figure 3). These 4 proteins were chosen as candidate liver metastasis-correlated proteins for the next clinicopathological analysis stage, which evaluated the correlationship between the expression of the candidate proteins by IHC and the clinicopathological factors in the surgically resected cases with PanNEN.

### Clinicopathological analysis stage

#### Patients’ characteristics

Of 118 PanNEN patients who received surgical resection between 1994 and 2016 at Tohoku University Hospital, 70 patients, excluding 48 without available formalin-fixed paraffin-embedded (FFPE) samples, were examined (Figure 1). Table 3 shows the clinicopathological background of the patients. The expression of the candidate proteins in PT was evaluated in patients with or without liver metastasis (Supplementary Figure 1). Synchronous and metachronous liver metastasis was seen in 8 (11.4%) and 12 cases (17.1%), respectively. No liver metastasis was observed in 50 cases (71.4%). High-grade in the WHO 2017 grade, Ki-67 labeling index, mitotic count, tumor size, vascular and lymphatic invasion rates and stage (European Neuroendocrine Tumor Society: ENETS) were significantly higher in the synchronous and metachronous metastasis groups (WHO 2017 grade: p = 0.002; Ki-67: p = 0.003; mitotic count: p = 0.017; tumor size: p = 0.001; vascular invasion: p < 0.001; lymphatic invasion p = 0.002; stage: p < 0.001).

**Table 3:** Patient characteristics in the clinicopathological analysis stage

Factors	Synchronous liver metastasis (n = 8)	Metachronous liver metastasis (n = 12)	Non-liver metastasis (n = 50)	*p* value
Gender (male: female)	5: 3	7: 5	23: 27	0.595
Age (median±SD)	51±15.5	53±12.8	58±17.9	0.478
Function type NF/ insulinoma/ gastrinoma/ glucagonoma (%)	7/0/1/0 (87.5/0/12.5/0)	8/ 2/ 1/ 1 (66.7/ 16.7/ 8.3/ 8.3)	33/ 15/ 1/ 1 (66.0/ 30.0/ 2.0/ 2.0)	0.054
WHO 2017 Grade G1/ G2/ G3 (%)	0/5/3 (0/62.5/37.5)	4/ 6/ 2 (33.3/ 50.0/ 16.7)	27/ 22/ 1 (54.0/ 44.0/ 2.0)	**0.002**
Ki-67 (median, range)	9.0 (2.0–30.5)	9.7 (1.1–80.0)	2.6 (0.1–25.2)	**0.003**
Mitotic count (median, range)	2 (1–7)	1 (0–8)	1 (0–14)	**0.017**
Tumor size (mm, median, range)	48 (14–75)	27 (10–150)	18 (6–70)	**0.001**
Lymph node metastasis Positive: Negative (positive rate, %)	3: 5 (37.5)	3: 9 (25.0)	6: 44 (12.0)	0.100
Vascular invasion Positive: Negative (positive rate, %)	8: 0 (100.0)	9: 3 (75.0)	15: 35 (30.0)	**<0.001**
Lymphatic invasion Positive: Negative (positive rate, %)	5: 3 (62.5)	7: 5 (58.3)	9: 41 (18.0)	**0.002**
Stage (ENETS) I: II: III: IV	0: 0: 0: 8	3: 6: 3: 0	23: 21: 6: 0	**<0.001**
Follow up period (Months, median, range)	75.4 (48.4–146.3)	54.8 (14.5–157.5)	57.6 (6.4–239.0)	0.206
Expression in PT; ANXA6 (%)	6 (75.0)	11 (91.7)	31 (62.0)	0.157
Expression in PT; CNPY2 (%)	7 (87.5)	10 (83.3)	19 (38.0)	**0.001**
Expression in PT; RAB11B (%)	5 (62.5)	6 (50.0)	25 (50.0)	0.864
Expression in PT; TUBB3 (%)	2 (25.0)	8 (66.7)	31 (62.0)	0.126

**Figure 4:**
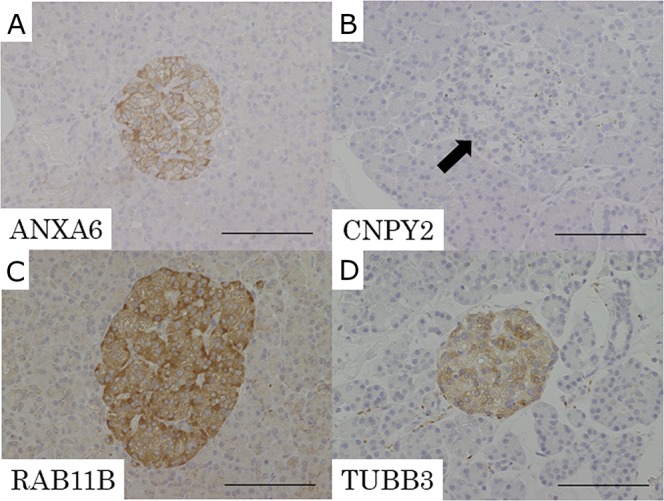
Representative pictures of IHC for the candidate proteins in normal pancreatic tissue **(A)** ANXA6, **(B)** CNPY2, **(C)** RAB11B, **(D)** TUBB3. Expression of CNPY2 was negative in normal islet cells (B: arrow). ANXA6, RAB11B and TUBB3 had positive expression in normal islet cells. Expression of ANXA6, CNPY2, RAB11B and TUBB3 was not detected in normal exocrine tissue of the pancreas. Scale bars indicate 100 μm.

In the IHC analysis, normal islet cells had negative expression of CNPY2 and positive expression of ANXA6, RAB11B and TUBB3 (Figure 4). The expression of these candidate proteins was not detected in normal exocrine tissue of the pancreas (Figure 4).

#### Correlationship between expression of candidate proteins and clinicopathological factors

In the immunohistochemical analysis for the candidate proteins, the positive expression rate of CNPY2 was significantly higher in both the synchronous and metachronous liver metastasis groups (p = 0.001). The WHO2017 histological grade was significantly correlated with the expression of CNPY2 (NET G1: 45.2%, NET G2: 48.5%, NET G3: 100.0%, p = 0.044) and RAB11B (NET G1: 35.5%, NET G2: 57.6%, NET G3: 100.0%, p = 0.010) (Supplementary Table 1). RAB11B positive cases were significantly higher in the Ki-67 labeling index compared with the negative cases (p = 0.005) (Supplementary Table 2). None of the immunostained proteins was correlated with the tumor size.

#### Liver recurrence-free survival and multivariate analysis

**Figure 5:**
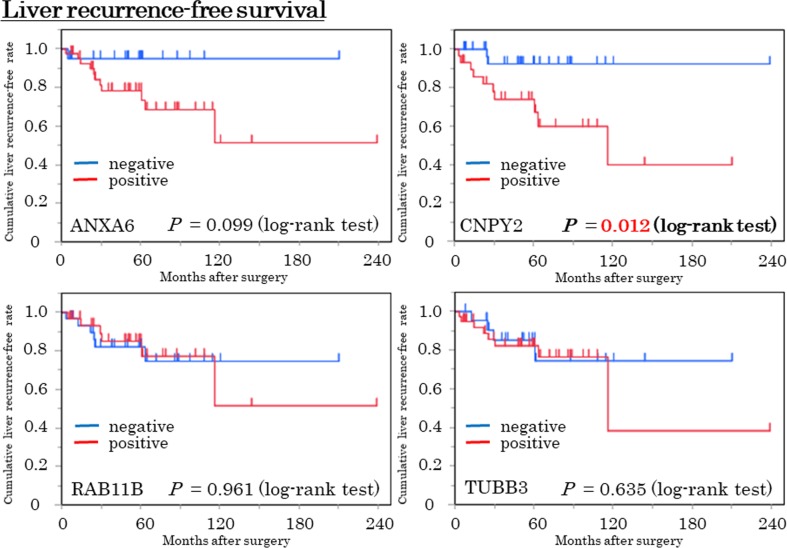
Liver recurrence-free survival (Liver RFS) curve in the expressions of the 4 candidate proteins by the Kaplan-Meier method Blue and red lines indicate negative and positive expressions, respectively. Patients who were CNPY2 positive were significantly poorer in liver RFS compared with CNPY2 negative patients (p = 0.012, log-rank test). Patients with ANXA6 positive tended to be poorer, compared with ANXA6 negative patients (p = 0.099, log-rank test).

To evaluate overall survival (OS) and liver recurrence-free survival (RFS) after surgical resection for primary pancreatic tumors, 62 cases without synchronous liver metastasis were investigated. OS and liver RFS were compared between the positive and negative expression of the candidate proteins. There was no significant correlation between OS and any of the candidate proteins (Supplementary Figure 2). Liver RFS was significantly poorer in the CNPY2 positive patients than in those who were negative (10-year liver RFS; 39.8% vs. 92.3%, p = 0.012). Also, liver RFS tended to be poorer in the ANXA6 positive patients than in the negative patients (10-year liver RFS; 51.4% vs. 95.0%, p = 0.099) (Figure 5). The expression of RAB11B and TUBB3 had no correlationship with liver RFS.

Predictive factors for liver RFS were examined by Cox proportional hazard analysis. The cut-off values of the Ki-67 labeling index and tumor size associated with liver RFS were investigated by receiver operating characteristic (ROC) curve analysis. The cut-off values of the Ki-67 labeling index and tumor size were 8.2% (area under the curve (AUC) = 0.708, p = 0.005), and 42 mm (AUC = 0.671, p = 0.010), respectively. As shown in Table 4, significant factors by univariate analysis included Ki-67 ≥ 8.2% (p = 0.009), tumor size ≥ 42 mm (p = 0.007), vascular invasion (p = 0.009), lymphatic invasion (p = 0.012) and CNPY2 positive (p = 0.010). ANXA6 was likely to be a predictive factor in the univariate analysis (p = 0.064). Significant factors in the univariate analysis were investigated for the multivariate analysis. In the multivariate analysis, the independent predictors of liver RFS were CNPY2 positivity (hazard ratio (HR): 6.19, 95% confidence interval (95% CI): 1.47–42.79, p = 0.011) and tumor size ≥ 42 mm (HR: 4.63, 95% CI: 1.03–23.23, p = 0.045).

**Table 4:** Univariate and multivariate analysis of clinicopathological factors and the candidate proteins in liver RFS

Factors (n = 62)	Univariate analysis		Multivariate analysis	
	HR (95%CI)	*p* value	HR (95%CI)	*p* value
Function: NF vs. functioning	1.39 (0.76–2.65)	0.293		
WHO 2017 Grade: G2/G3 vs. G1	2.03 (0.64–7.62)	0.235		
WHO 2017: Grade G3 vs. G1/G2	3.50 (0.52–13.91)	0.117		
Ki-67 ≥ 8.2%: yes vs. no	**4.76 (1.51–16.18)**	**0.009**	0.95 (0.20–5.36)	0.950
Tumor size ≥ 42 mm: yes vs. no	**5.80 (1.69–18.46)**	**0.007**	**4.63 (1.03–23.23)**	**0.045**
Lymph node metastasis Positive vs. Negative	1.43 (0.31–4.90)	0.610		
Vascular invasion Positive vs. Negative	**4.96 (1.48–22.38)**	**0.009**	1.11 (0.14–7.94)	0.921
Lymphatic invasion Positive vs. Negative	**4.37 (1.39–14.81)**	**0.012**	3.84 (0.90–17.54)	0.069
Stage (ENETS): III/IV vs. I/II	1.43 (0.31–4.90)	0.610		
ANXA6	4.77 (0.92–87.22)	0.064		
CNPY2	**5.60 (1.47–36.48)**	**0.010**	**6.19 (1.47–42.79)**	**0.011**
RAB11B	0.97 (0.30–3.11)	0.961		
TUBB3	1.34 (0.42–5.07)	0.631		

## DISCUSSION

Liver metastasis of NETs is an independent prognostic factor, regardless of the primary organ [3]. Liver metastasis was found at the initial diagnosis in 40–45% of the patients with NETs of the small intestine, pancreas, and colon [3]. Although the development of new drug treatments is needed to improve the prognosis in liver metastasis of PanNEN, the mechanisms of liver metastasis of PanNEN remain unclear.

It is important to detect some specific novel proteins that are strongly expressed or inhibited in liver metastasis of PanNEN for clarifying the mechanism, preventing the onset and improving treatment. For this purpose, we compared the protein expression between the primary tumor and the paired liver metastasis using proteomic analysis in the present study. There has been no study about discovery of novel liver metastasis-correlated proteins of PanNEN by proteomic analysis. However, we previously succeeded in detecting new biomarkers of pancreatic and bile duct cancer using this analysis [10, 11]. Therefore, we considered that it could also be a useful tool for the detection of liver metastasis-related proteins of PanNEN.

At first, we selected 10 proteins from among identified proteins in PT and/or LT as candidates that showed more than a 2 fold difference in the expression levels between PT and LT. Among these 10 proteins, ANXA6, CNPY2, RAB11B and TUBB3, all of which showed higher expression in LT, were focused on according to the confirmation of protein expression by IHC. No characteristic heterogeneous staining was found in IHC for these candidate proteins. Of the four proteins, we found that CNPY2 could be novel liver metastasis-correlated proteins of PanNEN. The present study demonstrated that the expression of CNPY2 and tumor size were independent predictors of liver RFS. Several previous studies revealed that the histological grade, Ki-67 labeling index, tumor size, vascular invasion and lymphatic invasion were correlated with recurrence and survival [17–19]. While positive data of liver metastasis-correlated proteins could be seen, the overall survival indicated no correlation with the expression of CNPY2 in this study. This might be because the population of the cohort was small and thus further studies on CNPY2 in a larger cohort are needed.

The function of CNPY2, previously called MIR-interacting saposin-like protein (MSAP), putative secreted protein Zsig9 (ZSIG9) or transmembrane protein 4 (TMEM4), is still unknown. There have been only a few published reports assessing the function of CNPY2. Guo et al. demonstrated that CNPY2 was regulated by hypoxia inducible factor (HIF)-1 alpha in human smooth muscle cells (SMC) and was a secreted angiogenic growth factor that promotes SMC migration, proliferation, and tissue revascularization by p53 inactivation [20, 21]. It has been reported that CNPY2 is related to esophageal squamous cell carcinoma (ESCC) [22], colorectal cancer (CRC) [23] and renal cancer [24]. No report on other malignancies including NETs has been published. In ESCC, patients with high expression of CNPY2, evaluated by IHC, showed significantly poor OS and RFS compared to those with low expression [22]. CNPY2 promoted tumor development through regulating p53 expression in CRC and renal cancer cells [23, 24]. Yan et al. demonstrated that knockdown of CNPY2 reversibly increased p53 activity using the colorectal cancer cell line HCT116 and suggested that CNPY2 plays a critical role in CRC development by enhancing cell growth, migration, and angiogenesis and by inhibiting apoptosis through negative regulation of the p53 pathway [23]. P53 has been reported to show abnormal immunolabeling for pancreatic neuroendocrine carcinoma (NEC), but not for PanNEN [25, 26]. Further studies are needed to examine the relationship between CNPY2 and p53 in PanNEN. The current study suggests that CNPY2 has potential as a biomarker for liver metastasis of PanNEN. CNPY2, which is a secreted protein, might have potential as a blood biomarker in liver metastasis of PanNEN because CNPY2 was detectable in murine blood plasma by enzyme-linked immunosorbent assay (ELISA) [35].

ANXA6 belongs to a family of calcium-dependent membrane and phospholipid binding proteins. ANXA6 participates in membrane and cytoskeleton organization, cholesterol homeostasis, membrane trafficking, cell adhesion and signal transduction [27, 28]. In malignancies, ANXA6 has been reported to play a role as a tumor suppressor in melanoma [29], breast cancer [30, 31] and gastric cancer [32]. In contrast, ANXA6 has been suggested to be a tumor promoter in cervical cancer [33], large cell lymphoma [28] and the stroma of pancreatic ductal adenocarcinoma [34]. There has been no report on the expression of ANXA6 in NETs. The results of the current study showed that PanNEN patients with ANXA6 expression were likely to have poor liver RFS. ANXA6 might also play a promotive role in the liver metastasis of PanNEN.

## MATERIALS AND METHODS

### Discovery stage

#### Patient selection and formalin-fixed paraffin-embedded (FFPE) tissue samples for proteomic analysis

One hundred and eighteen patients with PanNEN who underwent surgical resection between 1994 and 2016 at Tohoku University Hospital were examined. Twenty cases had synchronous or metachronous liver metastasis. Of these cases with liver metastasis, 7 cases had available FFPE samples of both PT and LT of PanNEN in an identical case, and were applied for proteomic analysis as the discovery set (Figure 1). Protein expression was compared between PT and LT by proteomic analysis. This study was approved by the Institutional Review Board of Tohoku University (the reference number 2017-1-437). Table 1 shows the patients’ characteristics at the discovery stage. The histological grade was determined according to the World Health Organization (WHO) 2017 of PanNENs [36]. The cohort contained patients with 5 synchronous and 2 metachronous liver metastases. In the histological grade, NET G2 and NET G3 included 4 and 3 cases, respectively. No patients with NEC G3 were included in the cohort. One case was gastrinoma, and the others were non-functioning PanNEN. Four patients died of the PanNEN. There was no patient with multiple endocrine neoplasia (MEN) in the cohort.

#### Laser microdissection (LMD) and protein extraction

LMD and protein extraction were performed as previously described [9, 10]. Briefly, 10 μm sections of the FFPE tissue samples were attached to DIRECTOR^™^ slides (Expression Pathology, MD, USA). After de-paraffinization with xylene, the samples were rehydrated with ethanol, stained with hematoxylin and then air-dried. Using a Leica LMD7000 (Leica Microsystems GmbH, Wetzler, Germany), approximately 30,000 tumor cells (8 mm^2^) were collected into the cap of a 0.2 ml polymerase chain reaction (PCR) tube. Peptide extraction was performed with a Liquid Tissue^™^ MS Protein Kit (Expression Pathology) according to the manufacturer's instructions [37].

#### Nano HPLC/MS/MS analysis for proteomics

The dried peptide extracts (2–4 μg) were dissolved together in 20 μl sample solution [5% acetonitrile and 0.1% trifluoroacetic acid (TFA)]. Each sample (10 μl) was injected into an EasynLC-1000 system (Thermo Fisher Scientific Inc., MA, USA) that was connected to an EASY-Spray column (25 cm length × C18 ODS 75 μm, Thermo Fisher Scientific Inc.). Peptides were eluted with a 180 min gradient of 4% to 25% solvent B (0.1% formic acid in acetonitrile, v/v) in solvent A (0.1% formic acid in water, v/v) at a flow rate of 300-400 nl/min. Peptides were then ionized and analyzed by a fusion mass spectrometer (Thermo Fisher Scientific Inc.) using a nano-spray source. High-resolution full scan MS spectra (from *m/z* 400–2,000) were acquired in the Orbitrap with resolution (R = 120,000 at *m/z* 400) and lock mass enabled (*m/z* at 445.12003 and 391.28429), followed by MS/MS fragmentation of the most intense ions for 3 sec in the linear ion trap with a collisionally activated dissociation (CID) energy of 35%. The exclusion duration for the data-dependent scan was 0 s, and the isolation window was set at 10.0 *m/z*.

The MS/MS data were analyzed by sequence alignment using variable and static modifications by Mascot and Sequest algorithms. The protein database utilized was UniProt. The specific parameters for protein sequence database searching included oxidation (M), deamination (N, Q), acetylation (N-term.), and pyroglutamation (E) as variable modifications, and carbamidomethylation (C) as a static modification. Other parameters used in the data analysis were: two allowed missing cleavages, a mass error of 10 ppm for precursor ions, and 0.8 Da for fragment ions. Charge states of +2 to +4 were considered for parent ions. If more than one spectrum was assigned to a peptide, only the spectrum with the highest Mascot score was selected for manual analysis. All peptides identified with a peptide score of Mascot > 20, and Sequest > 0.8 were manually examined using the protocol described previously [38].

#### Semi-quantitative comparison by spectral counting method

We compared the protein expression across all tissue samples from the results of the shotgun proteomics using the label-free spectral counting method, as previously described [9, 10, 14]. Fold changes in expressed proteins on a base 2 logarithmic scale were evaluated with the protein ratio from the spectral counting (Rsc) [39]. Relative abundances of the identified proteins were investigated by the normalized spectral abundance factor (NSAF) [40]. Comparisons of protein expression between the pancreatic and liver tumors were evaluated with the spectral index (SpI), ranging from −1 to +1 [41]. SpI values close to 0 indicated nearly equal relative peptide abundance in the compared groups. Candidate proteins were selected to satisfy Rsc > 1 or < −1 and p value < 0.05 in *G*-test [42] and were narrowed down using NSAF and SpI values.

#### Immunohistochemistry

IHC was performed as previously described [9, 10, 43]. Briefly, 4-μm FFPE tissue sections de-paraffinized with xylene and rehydrated with ethanol solutions and distilled water were heated in citrate acid buffer (10 mmol/l citric acid, pH 6.0) at 121°C for 5 min with an autoclave or microwave oven for 15 min in citrate acid buffer (10 mM citric acid, pH 6.0) for antigen retrieval. Antibodies of ANXA6 (ab31026, dilution 1:25, Abcam, Cambridge, UK), CNPY2 (ab181217, dilution 1:50, Abcam), HIST1H3A (ab174712, dilution 1:20, Abcam), Microtubule-associated protein RP/EB family member 1 (MAPRE1) (ab117821, dilution 1:250, Abcam,), Ras-related protein Rab-11A (RAB11A) (ab180778, dilution 1:50, Abcam), RAB11B (PA5-31348, dilution 1:100, Thermo Fisher Scientific Inc., MA, USA), Signal peptidase complex catalytic subunit (SEC11A) (ab174794, dilution 1:100, Abcam), Tropomyosin alpha-1 chain (TPM1) (ab55915, dilution 1:50, Abcam), TUBB3 (ab18207, dilution 1:1000, Abcam), Vesicle-associated membrane protein 3 (VAMP3) (10702-1-AP, Proteintech, Inc., IL, USA) were used as the primary antibodies. The sections were incubated over night at 4°C with one of the primary antibodies. After blocking endogenous peroxidase with methanol containing 0.3% hydrogen peroxidase, the labeled antigens were identified by an EnVision^+^ Sytem-HRP (DAKO, Glostrup, Denmark) and visualized by 3, 3’-diaminobenzidine tetrahydrochloride as a chromogen. The sections were lightly counterstained with hematoxylin.

Two of the authors (M.S., T.S.) completely reviewed all slides of the immunostained sections in each sample and classified the cases into two groups. We defined ≥ 10% tumor cells with staining of protein as positive expression, and < 10% tumor cells as negative expression. PT and LT samples in the discovery stage and pancreatic tumors of PanNEN in all cases except for those without available FFPE samples in the next clinicopathological analysis stage were examined by IHC for candidate proteins.

### Clinicopathological analysis stage

Of 118 patients with PanNEN, pancreatic tumors of 70 patients with available FFPE tissue were evaluated by IHC for protein expression of the candidate proteins. One patient with MEN type1 was included in the analyzed cohort. The duration of follow-up was calculated from the date of initial surgery to the date of death or last follow-up. Liver RFS was defined as the length of time that patients survived without initial liver metastasis after resection of the primary pancreatic tumor. Eight patients with synchronous liver metastasis were excluded from the analysis of OS and liver RFS (Figure 1). Liver metastasis-correlated factors were identified from clinicopathological factors including candidate proteins by univariate and multivariate analyses with a Cox proportional hazards model.

### Statistical analysis

JMP software version 13.0 (SAS Institute, NC, USA) was used for all analyses. Significance was calculated using Fisher's exact test, Pearson's χ^2^-test for categorical variates and Mann–Whitney U test, Kruskal–Wallis test for continuous variates. In the univariate analysis, OS and liver RFS rates were calculated by the Kaplan–Meier method and compared using the log-rank test. Univariate and multivariate analyses were performed using a Cox proportional hazards model to examine potential factors influencing the liver RFS. P < 0.05 was considered statistically significant.

## CONCLUSIONS

We have demonstrated for the first time that CNPY2 is a novel liver metastasis-correlated factor of PanNEN. Also, ANXA6 might affect the liver metastasis of PanNEN. Further studies are needed to elucidate the functions of CNPY2 and ANXA6 in PanNEN.

## SUPPLEMENTARY MATERIALS FIGURES AND TABLES



## Abbreviations

AUC: area under the curve
ANXA6: Annexin A6
CID: collisionally activated dissociation
CNPY2: the canopy FGF signaling regulator 2
CRC: colorectal cancer
ENETS: European Neuroendocrine Tumor Society
ESCC: esophageal squamous cell carcinoma
FFPE: formalin-fixed paraffin-embedded
HIF-1: hypoxia inducible factor −1
HIST1H3A: Histone H3.1
IHC: immunohistochemistry
KEGG: Kyoto Encyclopedia of Genes and Genomes
LC-MS/MS: liquid chromatography-tandem mass spectrometry
LMD: laser capture microdissection
LT: metastatic liver tumor
MAPRE1: microtubule-associated protein RP/EB family member 1
MEN: multiple endocrine neoplasia
MRLC: myosin regulatory light chain
MS: mass spectrometry
MSAP: MIR-interacting saposin-like protein
NET: neuroendocrine tumors
NEC: neuroendocrine carcinoma
NSAF: the normalized spectral abundance factor
OS: overall survival
PanNEN: pancreatic neuroendocrine neoplasm
PT: primary pancreatic tumor
RAB11A: Ras-related protein Rab-11A
RAB11B: Ras-related protein Rab-11B
RAB25: Ras-related protein Rab-25
RFS: recurrence-free survival
ROC: receiver operating characteristic
Rsc: the protein ratio from spectral counting
PCR: polymerase chain reaction
SEC11A: signal peptidase complex catalytic subunit
SEER: the surveillance, epidemiology, and end results
SpI: spectral index
TMEM4: transmembrane protein 4
TFA: trifluoroacetic acid
TPM1: tropomyosin alpha-1 chain
TUBB3: tubulin beta-3 chain
VAMP3: Vesicle-associated membrane protein 3
WHO: World Health Organization
ZSIG9: putative secreted protein Zsig9
