# Supplementary material for: A novel liver metastasis-correlated protein of pancreatic neuroendocrine neoplasm (PanNEN) discovered by proteomic analysis
Source: Oncotarget. 2018 May 11;9(36):24291–303. doi: 10.18632/oncotarget.25110 (PMC5966250; doi:10.18632/oncotarget.25110)
Supplement: Supplementary file 1 [file oncotarget-09-24291-s001.pdf]

# A novel liver metastasis-correlated protein of pancreatic neuroendocrine neoplasm (PanNEN) discovered by proteomic analysis

## SUPPLEMENTARY MATERIALS

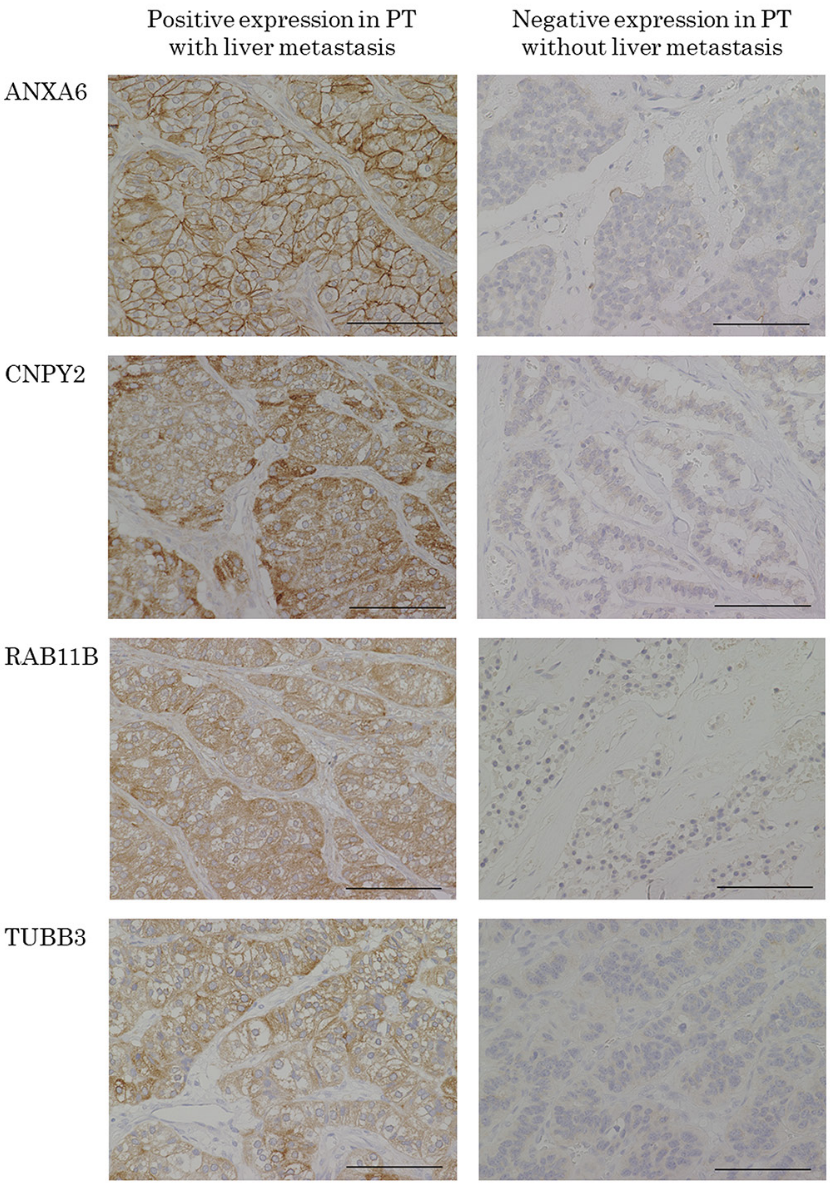


**Supplementary Figure 1: Representative pictures with high magnification of IHC for the candidate proteins.** Pictures of positive and negative expression were in PT with and without liver metastasis, respectively. Scale bars indicate 100 µm.

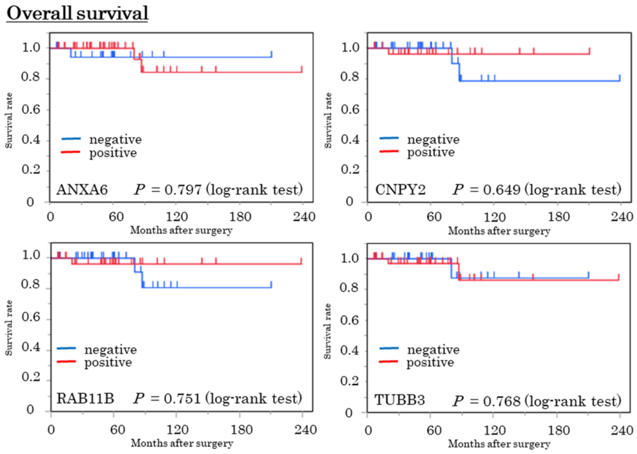


**Supplementary Figure 2: Overall survival (OS) in the expressions of the 4 candidate proteins by the Kaplan-Meier method.** Blue and red lines indicate negative and positive expressions, respectively. There was no significant correlation between OS and the expressions of the 4 candidate proteins.

**Supplementary Table 1: Expression of the candidate proteins in WHO 2017 Grade**

| Candidate proteins | G1 (n = 31) | G2 (n = 33) | G3 (n = 6) | *p* value |
|---|---|---|---|---|
| ANXA6 (Positive rate, %) | 67.7 | 66.7 | 83.3 | 0.715 |
| CNPY2 (Positive rate, %) | 45.2 | 48.5 | 100.0 | **0.044** |
| RAB11B (Positive rate, %) | 35.5 | 57.6 | 100.0 | **0.010** |
| TUBB3 (Positive rate, %) | 71.0 | 45.5 | 66.7 | 0.107 |

**Supplementary Table 2: Expression of the candidate proteins in Ki-67 or tumor size**

| Candidate proteins | IHC | Ki-67 (%, average±SD) | *p* value | Tumor size (mm, average±SD) | *p* value |
|---|---|---|---|---|---|
| ANXA6 | Positive (n = 48) | 7.1±8.1 | 0.404 | 27.2±24.4 | 0.569 |
| | Negative (n = 22) | 7.6±16.7 | | 26.5±16.6 | |
| CNPY2 | Positive (n = 36) | 10.3±14.9 | 0.127 | 28.3±18.5 | 0.282 |
| | Negative (n = 34) | 4.1±3.8 | | 25.6±25.6 | |
| RAB11B | Positive (n = 36) | 10.5±14.7 | **0.005** | 25.7±16.8 | 0.963 |
| | Negative (n = 34) | 3.8±4.1 | | 28.4±26.8 | |
| TUBB3 | Positive (n = 41) | 6.8±13.3 | 0.265 | 23.0±13.2 | 0.349 |
| | Negative (n = 29) | 7.9±8.0 | | 32.7±30.0 | |
